# Risk of cardiovascular disease? A qualitative study of risk interpretation among patients with high cholesterol

**DOI:** 10.1186/1471-2296-14-137

**Published:** 2013-09-16

**Authors:** Pia Kirkegaard, Adrian Edwards, Mette Bech Risør, Janus Laust Thomsen

**Affiliations:** 1 Research Unit for General Practice & Research Centre for Cancer Diagnosis in Primary Care, Aarhus University, Aarhus, Denmark; 2 School Public Health, Section for General Practice, Aarhus University, Bartholins Allé 2, DK-8000 Aarhus, C, Denmark; 3 Cochrane Institute of Primary Care and Public Health, School of Medicine, Cardiff University, Cardiff, UK; 4 General Practice Research Unit, Institute of Community Medicine, University of Tromsø, Tromso, Norway; 5 Research Unit for General Practice, Institute of Public Health, University of Southern Denmark, Odense, Denmark

**Keywords:** Risk perception, Cardiovascular disease, High cholesterol, Qualitative research

## Abstract

**Background:**

Previous studies have shown the importance of paying attention to lay peoples’ interpretations of risk of disease, in order to explain health-related behavior. However, risk interpretations interplay with social context in complex ways. The objective was to explore how asymptomatic patients with high cholesterol interpret risk of cardiovascular disease.

**Methods:**

Fourteen patients with high cholesterol and risk of cardiovascular disease were interviewed, and patterns across patient accounts were identified and analysed from an ethnographic approach.

**Results:**

Information from the general practitioner about high cholesterol and risk of cardiovascular disease was reinterpreted in everyday social life. The risk associated with fatty foods was weighed against the pleasures of social and cultural events in which this type of food was common and cherished.

A positive mindset was applied as a strategy to lower the risk of having high cholesterol, but knowledge about risk was viewed as a cause of anxiety and self-absorption, and this anxiety made the body susceptible to disease, hampering the chances for healthy life.

**Conclusion:**

Interpretations of high cholesterol and risk of cardiovascular disease are embedded in social relations and everyday life concerns. This should be addressed in general practice in preference-sensitive cases about risk-reducing medication.

**Trial registration:**

ClinicalTrials.gov: NCT01187056

## Background

Cardiovascular disease accounted for 30% of all deaths in 2008 and it is the number one cause of death worldwide [1]. High cholesterol is a well-defined risk factor for cardiovascular disease, and it is associated with unhealthy diet and lack of exercise [2]. High cholesterol can be lowered by lifestyle change combined with cholesterol-reducing medication [3,4].

However, up to half of all patients prescribed with cholesterol-reducing medication stop treatment within 6 months, with a further decline after one year [5,6]. The decline is most prominent in patients with no bodily perceptible symptoms of cardiovascular disease. Short-term cholesterol-reducing medications have no documented health benefits and discontinuation of cholesterol-reducing medication or on/off use is unwanted from individual and health service perspectives [7].

The reasons for the non-adherence/discontinuation of cholesterol-reducing medication have been explored [8-12]. The studies report that epidemiological knowledge about risk of disease, conveyed by doctors, may be little understood by patients. Efforts have been made to reduce the proportion of patients who feel uncertain about the decision to engage in medical preventive treatment for as long as the general practitioner (GP) prescribes, but results about the effect on adherence remain inconclusive [13]. However, everyday life experiences, risk interpretations and social negotiations, outside the doctor’s consultation, have been little explored. Anthropological and sociological studies have shown the importance of exploring people’s interpretations of health and risk as part of ongoing social processes, in which different rationalities for action are used in interpretations about different matters [14-20]. Decisions to continue or stop risk-reducing medication are embedded in everyday life concerns which form a background for the patients’ deliberations about risk of the disease and strategies to approach that risk.

The aim of this study was to explore how cholesterol-reducing medication and risk of cardiovascular disease are interpreted by asymptomatic patients with high cholesterol. The following research questions were addressed: What is the relationship between risk of cardiovascular disease and everyday life concerns? How is the risk of having high cholesterol weighed against other risks or concerns? What is the role of the GP in relation to this?

## Methods

### Participants and recruitment

The qualitative study presented in this paper is a part of the RISAP study – A Complex Intervention in Risk Communication and Shared Decision-Making in General Practice [21,22]. The aim of the RISAP study was to develop risk information for patients at high risk of cardiovascular disease, and to evaluate the effect on the number of prescribed cholesterol-reducing medication.

Fourteen patients were recruited through five GPs who had participated in focus group discussions in the development of the RISAP study. The GPs were asked to provide the interviewers (two trained social anthropologists) with the name and address of the last patients who had entered their practices and fulfilled the following criteria: 1) patients who had high cholesterol and high or very high risk of cardiovascular disease according to the Danish guidelines for prevention of cardiovascular disease in general practice [23], 2) patients who had received information about cholesterol-reducing medication as a preventive treatment option, and 3) patients who had no manifest cardiovascular disease or symptoms of it. Fifteen patients were contacted by a letter from the researchers with information about the study, and 14 patients – 8 women and 6 men – gave oral consent to participate in an interview without renumeration (Table 1). They were interviewed individually (n=8) or with their spouses (n=6), and the interviews were supplemented with fieldnotes about the interviews. Each interview lasted 1-2 hours and took place in the patients’ homes. Twelve out of 14 patients reported to take cholesterol-reducing medication. The sample included patients from both primary and secondary prevention of cardiovascular disease, but at the time of the interview, none of them had symptoms which they interpreted as related to high cholesterol or to cardiovascular disease. The participants appear under pseudonyms in the paper.

**Table 1:** Patient characteristics

**Alias**	**Age**	**Marital status**	**Co-morbidity**
Ivan	61	Widower	Pre-diabetes
Judith	60	Married	None
Beth	65	Married (Kent)	None
Kent	65	Married (Beth)	None
Christian	67	Married (Ulla)	None
Ulla	64	Married (Christian)	Diabetes
Mary	61	Married (Frank)	Familial hypercholesterolemia
Frank	62	Married (Mary)	None
Laura	66	Married	None
Christina	61	Married	Diabetes
Tom	61	Married	Pre-diabetes
Karen	70	Widow	Stroke
Ursula	24	Single	Familial hypercholesterolemia
Kurt	59	Married	Diabetes

The study was performed according to the Helsinki Declaration [24]. It was notified to the Danish Data Protection Agency and collection of data was handled according to their guidelines (journal no. 2007-41-1446). The study was exempted from obligation of notification for the Danish Scientific-Ethical Committee, but it followed the ethical code of American Anthropological Association [25]. The study was part of the RISAP study which was registered at ClinicalTrials.gov: NCT01187056.

### Analytic approach and data analysis

An ethnographic approach was used to support the interplay between theory, methods and data in the analytical process [26]. Firstly, the literature about risk perception of patients at high risk of future disease was reviewed by the first author, and social theories about risk perception in the studies were discussed among the researchers. Secondly, the theories helped frame questions for a semi-structured interview guide [27]. The following themes were included: experiences with risk of cardiovascular disease (what is it like to have it?); interpretations of risk of cardiovascular disease (what is it?), risk negotiations (what did the GP say to you about it, how did you respond, what did you tell your family and friends, and how did they respond?). Each interview was transcribed verbatim into text within few days after it was performed, and handwritten fieldnotes were typed up. The texts were put into a computer-based standard text editor with tracked changes and thus discussed among the researchers to jog the analytic process. The first interviews drew our attention to the fact that the patient accounts revolved explicitly around high cholesterol, and less about cardiovascular risk, and the interview guide was refined accordingly by adding questions about high cholesterol to the themes mentioned above. Thirdly, a preliminary analysis was agreed upon by the researchers, and finally, the identified themes were approached from theoretical perspectives to strengthen and contextualise the findings in literature about risk. We chose to use the notion of risk interpretation in the paper, in order to underline the focus on the social aspect of risk. The capital letters in parentheses refer to the quotes in Table 2.

**Table 2:** Quotes from participants unless otherwise indicated

A	Judith: We are at risk here in our part of the world, in our culture…. In other places, the risk is different – environmental pollution in India or Japan… that’s the way it is. It’s different. (…) Maybe it has something to do with our way of life in Denmark, you know, generally speaking. Many Danes have it [high cholesterol], don’t they. (…) We live in abundance here in Denmark, you know….anyway, I really watch what I eat, I mean I don’t eat that much sugar and fat.
A	Ursula: We just eat everytime we meet. Pizzas, shawarmas…when you meet in the city on a café, or you go out at night… and we always end up in a pizza bar somewhere at 3 a.m. Sure, it’s not that healthy for your body, but… I probably wouldn’t miss it.
A	Ulla: You meet for a cosy chat, have something to eat… We hold on to the South Jutish cakes [det sønderjyske kaffebord], seven soft cakes, seven hard cakes…. Sometimes up to 30 cakes…! […] Good, old-fashioned food for us.
A	Christina: My diabetes, I have to be careful […] Gravy and pork cracklings… It’s extra important to enjoy every bite, when you think about it. In the family…I mean, carrots and mineral water! [..?..] they would be very disappointed indeed.
B	Judith: no, I’m not really afraid of it [high cholesterol]. But I find it annoying […]. Then I think to myself “OK you’re not as young as you were”. You can’t… That’s how I think about it. Something will turn up as you get older. Something will turn up.
B	Karen: well, you don’t get younger as years go by, that’s it, really. [..] You go into ’repair mode’, something happens in your body and you won’t really feel it.
B	Beth: [about husband Kent] he plays in the Old Boys League, and that’s one way to do it, so you don’t get a heart attack sitting in front of the TV…. you’ll die on the football field (both laugh).
C	Ivan: I trust the experts. If they tell me, that it is the right thing to do [take medication] – then it is the right thing to do. And as he said to me, we cannot prolong your life. But you can still do something – maybe you can avoid a blood clot or something else. And then perhaps you could have a better quality of life. But of course, you can’t take pills for everything and then live 15 years longer. He said, that’s not the point. He said that the point is to reduce the numbers [cholesterol level]. That’s what it’s all about. Reducing the number.
C	Kurt: he said it was OK, no danger ahead, and the medication keeps it low. […] He keeps an eye on me, my blood pressure, the fat in the blood, all my numbers, so it won’t go too high. I can’t feel it, but they see it…the good and the bad fat in you. Not his fault I enjoyed my youth.
C	Laura: you live your own life, it’s no one’s responsibility in the end, you know. He just keeps an eye on you, the nurse keeps an eye… […] it was 6.2 [turns over pages of a printout from the lab] and now its below 3, see, all is in place. You still have to eat… properly, or the medication won’t work, for sure, I’m sure it won’t.
D	[Fieldnotes] Mary, Frank, married, retired early after a life of hard physical work. Mary, tiny woman, swears that she never eats sweet things, Frank, a sturdy man with a self-reported ‘sweet tooth’. Mary says her father died because his main coronary artery clotted, and her GP worried that Mary might die from the same thing, if she doesn’t take her cholesterol-reducing medication. In both Mary’s and Frank’s families, many members have died from blood clots, but Mary thinks she has ‘a certain kind of cholesterol’, different from that of Frank’s. [Quote] Mary: slim people get it too. One of our friends… he is quite slim. And his cholesterol is very high indeed. It has nothing to do with obesity.
D	Tom: No surprise, really, if your dad had a stroke at 40…. or if you were extremely overweight….then you can have someone stitch your lips together [laugh] but in those cases, medication is probably not even enough…. In the end, it’s different, really, when it’s not running in the family, and you live a healthy life and feel OK and fit. It’s not that complicated. […] You do what you can do, move your body, take the stairs […] I don’t think that good cholesterol is dangerous, only to some people.
E	[Fieldnotes] Kent, Beth, 65 years old, retired, unskilled labour. Beth’s cholesterol level is too high, takes medication. Kent had a health check 14 days ago.
	[Interview dialogue]
	Kent: Then, mine was really a bit too high, too.
	Interviewer: what did she [the GP] say to you?
	Kent: she said ‘it’s a bit too high.’ Then she said ‘what should we do about that?’ Then I said ‘we don’t do any damn thing about it’.
	Interviewer: what did she say then?
	Kent: you know, I eat a herring every day!
	Beth (interrupting) [she said] we should try to lose some weight at Christmas time.
	Kent: we’ll have to give it another go.
	Interviewer: so you’ll just try again?
	Kent: Yes! I won’t take medication to prevent disease.
	Interviewer: no..?
	Kent: I only want medication if something is wrong with me.
	Interviewer: what did she say, then? Why…?
	Kent: I don’t want a load of trash in me. I’m a natural kind of guy!
	[…]
	Interviewer: what if it turns out that you really need cholesterol-reducing medication?
	Kent: well, it has to be absolutely necessary. Because, as I said before, I won’t take medication to prevent disease. I’d rather have another herring.
	Beth: [laughing] well, another herring won’t necessarily lower it.
	Interviewer: but what do you think it [herring] will prevent?
	Kent: well, I don’t know.. what is it supposed to prevent?
	Beth: well, cardiovascular disease and… that’s it.
	Kent: yes, but..there’s nothing wrong with me. My blood pressure is fine. I have no diabetes even though it runs in the family. My mother’s brother whom I never knew, died of diabetes in the 30’ies or 40’ies or whenever the hell it was. 1940. And my mother died of diabetes. And my sister died of diabetes.
E	Christian: I said to him that….. I’m not really… fond of taking pills. I thought that we should wait and see.
	Interviewer: and this happened 5 or 6 years ago…?
	Christian: yes, and then later on the pursuaded me to try it.
	Interviewer: OK. What did he say?
	Christian: Well… he said that… I might get… blood clots and stuff if I didn’t do something.
	Interviewer: OK. Has anyone in your family had a blood clot?
	Christian: Yes. I have a brother who is paralysed on one side..
	Interviewer: Does it frighten you to think about that?
	Christian: oh no. Not the least.
	Interviewer: when did your GP tell you to try cholesterol-reducing medication?
	Christian: well, he wanted me to start it when I went to see him. Every single time for the last… 2 or 3 years. Until I gave in.
	Interviewer: what did he say to make you…[give in]?
	Christian: he said…well, actually I don’t remember, apart from him threatening me… saying that I could risk getting a blood clot too.
	Interviewer: did he tell you about your cholesterol level, what your numbers were, and stuff?
	Christian: well, I think it was about 6 at the time. And he wanted it to be lower than that.
	(…)
	Christian: It is unhealthy from my point of view. It is not natural. And I can’t see the point in taking it as long as I am not ill.
	Interviewer: but you do take it anyway…?
	Christian: I do, yes.
	Interviewer: Why?
	Christian: Well, uhm, I am under pressure.
	Ulla: come on, it’s not like that.
F	Kurt: In the end, all that fuss about blood sugar and fat on your belly… or bum, what is it… maybe you should start focusing a bit on other things. Appreciate your friends, take some time off, see all the good things you have in your life. Be a bit positive, for God’s sake, it won’t kill you…. Who invented medicine, that’s my point.
F	Kent: we are positive people. Positive people live longer. They really do […] Negative things come out of the blue. You shouldn’t pursue them.
F	[Field notes] Judith, 60, retired school teacher, adverse bodily sensations, interprets them as side-effects. Sensations connected to awareness about the potential side-effects as described in patient information leaflet.
	[Quote] Judith: I admit that it causes a lot of anxiety. It really does. All the different options. They cause a lot of anxiety. And perhaps sometimes it makes you focus a little bit too much on yourself. A bit anxious. You feel fine, right. That’s one aspect to it. It doesn’t make us happier – and perhaps happiness is the best life-prolonging medicine you can get…Don’t you think? [laugh].

## Results and discussion

Several participants suggested that high cholesterol was caused by a certain lifestyle with fatty foods and lack of exercise, and in some cases by genetic disposition (A,D). Most of the participants, however, reported to have healthy eating habits themselves - at least on weekdays. They stressed the significance of indulging fatty foods as part of a social event, i.e. eating together with family and friends and enjoying each other’s company while appreciating taste and composition and paying compliments to the host. The fatty foods, associated with cardiovascular risk by the participants, were only one aspect in the social events. The fatty foods were appreciated because of the well-known tastes and the traditions that surrounded them. Thus, the risk associated with fatty foods was weighed against the pleasures of social and cultural events in which this type of food was common and cherished.

Some partipants argued that a ‘positive’ or embracing attitude to life should accompany the focus on risk (F). An embracing attitude implied appreciation of pleasurable matters in everyday life, combined with less concern about natural bodily sensations or symptoms, which are well-known and common in older people (B). In this view, knowledge about risk caused anxiety and self-absorption, and this anxiety made the body susceptible to disease, hampering the chances for healthy life. Some participants emphasized a non-correlation between high cholesterol and the general health of the individual; if a person considered himself a ‘healthy person’ , high cholesterol represented minor risk or danger. In this view, a healthy life style could even outweigh the risk of having high cholesterol (E,F). However, most of the patients saw the cholesterol-reducing medication as as a valid response to high cholesterol, although they also stressed the individual responsibility to eat sensibly and exercise (C). Cholesterol expressed as a ’number’ was reported to be easy to relate to in the absence of a bodily perceptible sensations or symptoms. GPs were seen as brokers or interpreters of important and complex knowledge about the patient’s body, able to transform specific bodily properties into numeric indicators.

Thus, the analysis revealed an underlying interpretation of tight relations between body and mind, in which a slowly deteriorating body at increasing risk of cardiovascular disease was upheld by social events, positive mindsets, and cholesterol-reducing medication. High cholesterol was related to the social event of eating, and the social event was seen as a contributory reason to getting a high cholesterol level and maintaining it. Worry about high cholesterol would brace the medically defined risk of cardiovascular disease, but high cholesterol did not pose a high risk if maintaining a balance in physiological and mental health. Cholesterol-reducing medication could help lower the tangible numbers which were conveyed by the GP, who would provide reassurance and supervision.

The findings in this study suggest that information from the GP about high cholesterol and risk of cardiovascular disease was reinterpreted in everyday social life, where people navigate according to different ‘concerns’ (not to be confused with worry), using rationalities for action from different discourses [28]. In this theoretical perspective, knowledge and norms are only two in many premises for action; concerns related to experiences often guide action [29]. They are used to explain the apparent contradictions in people’s knowledge about a given matter, and their actions; e.g. people navigate between different concerns to overcome the discrepancy between knowledge about the right thing to do according to advice from health authorities (including the GP), and social relations that may imply risky behavior. Knowledge in this perspective is a social practice in which issues about cholesterol and cardiovascular disease is derived from multiple sources, including social experience in everyday life and medical/moral instructions about healthy living. This study indicates the importance of not reducing the patients’ reluctance towards the biomedical definition of high cholesterol as a risk for cardiovascular disease to non-adherence. Patients’ interpretations of high cholesterol make sense in a social context, and risk reduction of cardiovascular disease may not be acted upon as a primary concern par excellence. In all, this suggests a more open approach to the way patients make choices, in order to understand that risk of cardiovascular disease may not be a prominent concern in patients’ everyday lives.

It has been argued that GPs should address these concerns in order to improve communication and shared decision-making with patients about preventive treatment benefits and risks [30]. Felde and Elverdam have described how high cholesterol and risk of cardiovascular disease is embedded in a contextual rationality, contingent on the patient’s experience and social practice [31]. They urge GPs to pay attention to the way in which the patients interpret high cholesterol and apply meaning to it. They argue that general practice provides a particularly fruitful ground for addressing the patients’ risk interpretations, experiences and social practices.

Sångren et al. have investigated hypertensive patients at risk of cardiovascular disease [32]. They found that being diagnosed with hypertension constituted a biographical disruption, and patients with hypertension interpreted their condition as a chronic disease. In our study, we found no direct expressions of high cholesterol as a disease, and having high cholesterol was not interpreted as a major disruption in life. However, high cholesterol was in some ways acted upon as if it had a certain materiality – it was ‘real’ even though it was imperceptible, due to fact that it can be measured in numerical form.

### Limitation

The interview as method allows the researchers to gain access to the participants’ accounts of their actions and the meaning they apply to them, but not to the actions themselves except for the ones enacted during the interview [33]. Thus, it was not possible to observe ‘lived risk’ in the study, only to gain access to accounts about everyday life. Only five in 12 GPs responded to our request about recruiting participants for the interview study, and they provided us with one to four patients each. We assumed greater variance between the interviewed participants than between the GPs who recruited them, which was confirmed by the fact that each of the interviewed couples with high cholesterol had the same GP, and yet they had diverse interpretations of cardiovascular disease and the use of cholesterol-reducing medication.

## Conclusion

This study explored how asymptomatic patients with high cholesterol interpreted risk of cardiovascular disease. The research question was triggered by the observation that up to half of all asymptomatic patients with high cholesterol who initiate cholesterol-reducing medication, stop treatment after 6 months, with a further decline after 1 year. The study illustrates how patients accepted, opposed or pondered about the cholesterol-reducing medication as a proper response to lowering the risk of cardiovascular disease. It also showed that the GP plays a significant role in providing supervision of the cholesterol level. However, patients’ interpretations of high cholesterol and risk of cardiovascular disease are embedded in social relations and everyday life concerns which should be addressed by the GP during consultations about cholesterol-reducing medication as a treatment option.

## Competing interest

The authors declare that they have no competing interest.

## Authors’ contributions

PK and JT planned and drafted the study. PK, AE, and MR contributed to the study design and to the data analyses. PK and AGJ conducted the interviews. PK was responsible for the data analyses and for the paper draft. All authors read and approved the final manuscript.

## Pre-publication history

The pre-publication history for this paper can be accessed here:

http://www.biomedcentral.com/1471-2296/14/137/prepub
